# Acute toxicity of second generation HIV protease-inhibitors in combination with radiotherapy: a retrospective case series

**DOI:** 10.1186/1748-717X-6-25

**Published:** 2011-03-17

**Authors:** Alfred P See, Jing Zeng, Phuoc T Tran, Michael Lim

**Affiliations:** 1 Department of Neurosurgery, The Johns Hopkins Hospital, 600 N. Wolfe Street, Baltimore, MD 21287 USA; 2 Department of Radiation Oncology and Molecular Radiation Sciences, The Sidney Kimmel Comprehensive Cancer Center, The Johns Hopkins Hospital, 401 North Broadway, Baltimore, MD, 21231 USA; 3 Department of Oncology, The Sidney Kimmel Comprehensive Cancer Center, The Johns Hopkins Hospital, 401 North Broadway, Baltimore, MD, 21231 USA

## Abstract

**Background:**

There is little data on the safety of combining radiation therapy and human immunodeficiency virus (HIV) protease inhibitors to treat cancers in HIV-positive patients. We describe acute toxicities observed in a series of HIV-positive patients receiving modern radiation treatments, and compare patients receiving HIV protease inhibitors (PI) with patients not receiving HIV PIs.

**Methods:**

By reviewing the clinical records beginning January 1, 2009 from the radiation oncology department, we identified 29 HIV-positive patients who received radiation therapy to 34 body sites. Baseline information, treatment regimen, and toxicities were documented by review of medical records: patient age, histology and source of the primary tumor, HIV medication regimen, pre-radiation CD4 count, systemic chemotherapy, radiation therapy dose and fractionation, irradiated body region, toxicities, and duration of follow-up. Patients were grouped according to whether they received concurrent HIV PIs and compared using Pearson's chi-square test.

**Results:**

At baseline, the patients in the two groups were similar with the exception of HIV medication regimens, CD4 count and presence of AIDS-defining malignancy. Patients taking concurrent PIs were more likely to be taking other HIV medications (p = 0.001) and have CD4 count >500 (p = 0.006). Patients taking PIs were borderline less likely to have an AIDS-defining malignancy (p = 0.06). After radiation treatment, 100 acute toxicities were observed and were equally common in both groups (64 [median 3 per patient, IQR 1-7] with PIs; 36 [median 3 per patient, IQR 2-3] without PIs). The observed toxicities were also equally severe in the two groups (Grades I, II, III respectively: 30, 30, 4 with PIs; 23, 13, 0 without PIs: p = 0.38). There were two cases that were stopped early, one in each group; these were not attributable to toxicity.

**Conclusions:**

In this study of recent radiotherapy in HIV-positive patients taking second generation PIs, no difference in toxicities was observed in patients taking PIs compared to patients not taking PIs during radiation therapy. This suggests that it is safe to use unmodified doses of PIs and radiation therapy in HIV cancer patients, and that it is feasible to use PIs as a radiosensitizer in cancer therapy, as has been suggested by pre-clinical results.

## Background

### HIV and malignancies

Historically, HIV infection is associated with a much higher risk of specific cancers [1-4]. In particular, diagnosis of Kaposi sarcoma, non-Hodgkin lymphoma (NHL), or cervical cancer are considered acquired immunodeficiency syndrome (AIDS)-defining malignancies [5]. However, increasing effectiveness of anti-retroviral therapy (ART) has led to decreased mortality in Europe and North America from opportunistic infections and AIDS-defining malignancies [5-8], while mortality from non-AIDS-defining and non-HIV-associated cancers has been increasing [8,9].

Response to cancer therapy is also different in the HIV patient population. Initial reports found increased radiotoxicity in HIV patients receiving treatment for Kaposi sarcoma, cervical carcinoma, while there was no difference in adverse effects of radiation therapy for other malignancies [10,11]. Systemic glutathione deficiency [12], DNA repair deficiency, or cell cycle dysregulation may increase radiosensitivity [13-15]. However, radiation therapy remains a cornerstone of therapy in a number of cancers such as anal cancer [16], prostate [17], breast [18-20], and cervical cancer [16,21].

### Protease inhibitors in the treatment of HIV

PIs are anti-viral drugs that inhibit proteases, viral enzymes which cleave polyprotein precursors into mature viral proteins [22]. PIs are one class of anti-virals that is used as the 'base' in combination with two 'backbone' drugs for treatment of HIV, antiretroviral therapy (ART). There are currently ten PIs available; in chronological order of FDA approval, saquinavir, ritonavir, indinavir, nelfinavir, lopinavir, atazanavir, fosamprenavir (pro-drug of amprenavir, which is no longer available), tipranavir, and darunavir.

Although PIs act by inhibiting HIV aspartyl protease, they also have off-target effects. The entire class is associated with dysregulation of glucose and lipid metabolism due to homology between HIV-1 protease and various human proteins [23-26]. In addition, some PIs inhibit the phosphatidyl-inositol 3-kinase (PI3K)-Akt pathway, which is shared by numerous cell homeostasis pathways [27,28].

### Non-target effects of protease inhibitors

A number of PIs have been associated with anti-cancer activity [29]. Through PI3K-Akt and closely related pathways, PIs induce apoptosis of tumor cells [30-36]. Although PIs have been shown to directly effect tumor cell death, use of PIs has not reduced cancer risk in HIV patients, suggesting that PIs would not be clinically effective anti-cancer monotherapies [37]. Although ineffective alone, PIs synergize with other cancer therapies such as radiotherapy [38].

Initial studies suggested that nelfinavir upregulates vascular endothelial growth factor (VEGF) and downregulates hypoxia-inducible factor 1 alpha (HIF-1α). Although VEGF can increase tumor oxygenation, the HIF1-α hypoxia factor can mediate radiation resistance [39,40]. However, HIF-1α knockdown studies suggest that radiosensitivity induced by PIs is independent of HIF-1α [28,40-42]. In a number of cancers, resistance to radiotherapy is mediated by the PI3K-Akt pathway, suggesting an alternative mechanism of PI-induced radiosensitization [43-45]. Preclinical studies with nelfinavir in head-and-neck cancer [46] and non-small cell lung cancer [28] cell lines found downregulation of Akt to be associated with increased sensitivity to radiation.

Although PI-induced radiosensitization of cancers was shown to be independent of HIF-1α, PIs have been shown to induce systemic vascular stress [47]. Preclinical *in vivo *studies suggest that in addition to direct effect on the tumor cells, PIs may inhibit PI3K-Akt activation in tumor vasculature, suppressing hypoxia pathways and leading to reduced radiation resistance [48,49]. Other clinical reports also suggest that PIs and radiotherapy interact on tumor vasculature similar to the effects of radiation and bevacizumab, an anti-angiogenic antibody [50].

### Protease inhibitors and radiotherapy

A retrospective review (14 patients receiving PIs and 28 controls) did not find severe toxicities attributable to combination of PIs and radiotherapy for cancer in HIV+ patients [11,51-54]. There are ten prospective trials, nine of which are on-going (a phase II trial was terminated due to poor enrollment): five phase I studies, and four studies that have a phase II component. One published phase I trial in pancreatic cancer showed the following toxicites one of which was life-threatening: severe nausea and vomiting and increase in liver enzymes and bilirubin due to stent occlusion [55]. Given the inconclusive safety data on combining PIs and radiation therapy to treat cancer in HIV patients, we reviewed a series of HIV patients receiving radiation therapy for malignancies.

## Methods

### Patient identification

In accordance with a research protocol approved by the Institutional Review Board, patients were identified by review of clinical records from January 1, 2009-October 31, 2010 in the Department of Radiation Oncology at The Johns Hopkins Hospital. Patients were included if they had documented HIV infection and received radiation therapy at Johns Hopkins.

### Retrospective review

Medical records for included patients were reviewed for HIV medications, cancer diagnosis and stage, radiation therapy (site, dose, fractionation, completion or early stopping), age at time of radiation therapy, cancer chemotherapy, acute (< 6 weeks after end of radiation therapy) toxicities categorized by Common Toxicity Criteria for Adverse Events version 3.0 (CTCAE) grade. All patients receiving radiation therapy were evaluated at least once per week for treatment toxicity, and side effects were recorded prospectively in an electronic record system.

### Statistical analysis

Patients were categorized by type of malignancy (AIDS-defining, HIV-associated, non-HIV associated), taking non-PI HIV medications (yes/no), and by baseline CD4 count (< 50, <200, <500, 500). Toxicities were categorized by CTCAE grade. Differences between the groups were analyzed using Pearson's chi-square test with JMP 8.0 (SAS Institute Inc.). Statistical significance was defined as a Pearson's chi-square p-value < 0.05.

## Results

We retrospectively reviewed acute toxicities in a series of patients with a history of HIV infection and receiving radiation therapy; in this series, we compared patients who received concurrent PIs with patients who did not receive concurrent PIs. Eighteen patients received concurrent PIs and radiation therapy; one patient received radiation therapy for two different malignancies, and one patient received radiation for three recurrences of NHL. There were eleven patients with a history of HIV infection but not treated with PIs who received radiation therapy; one patient received three regimens of radiation therapy, twice for brain metastasis and once for testicular metastasis.

### Patient characteristics

Characteristics of patients receiving concurrent protease inhibitor are presented in Table 1 while characteristics of patients not receiving concurrent protease inhibitor are presented in Table 2. There were 34 total courses of radiation treatment delivered (21 with PIs, 13 without PIs) for a variety of histologies, including HIV-defining (0 with PIs; 3 [23%] without PIs), HIV-associated (11 [58%] with PIs; 5 [38%] without PIs), non-HIV-associated malignancies (8 [42%] with PIs, 5 [38%] without PIs), and non-malignancies (keloid scar and dural arteriovenous fistula with PIs, none without PIs). The median age was 50 (interquartile range [IQR] 47-56). The difference between the two groups in number of AIDS-defining malignancies almost reached statistical significance (p = 0.06), but the remainder of the malignancies (HIV-associated and non-HIV-associated) are not differently distributed in the two groups (p = 0.9). 29 cases had documented pre-treatment CD4 counts; 4 were <50 (4 [24%] with PIs), 13 were <200 (9 [53%] with PIs, 4 [33%] without PIs), and 21 were <500 (10 [59%] with PIs; 11[92%] without PIs). Patients taking PIs were more likely than patients not taking PIs to have a CD4 count≥500 (7 [41%] with PIs; 1 [8%] without PIs; p-0.006).

**Table 1:** Baseline data: patients receiving concurrent protease inhibitor

#	Cancer diagnosis	Age	Concurrent systemic therapy	Baseline CD4	Non-PI HIV regimen	PI
1a	Ductal carcinoma, breast T2N1M0	47	None	104	lamivudine, raltegravir	RTV, DRV
1b	SCC, anus T3N0M0	49	5-FU, mitomycin C	68	lamivudine, raltegravir	RTV, DRV
2	SCC, vulva T1bN1b, stage IIIa	26	cisplatin	1647	emtricitabine, tenofovir	RTV, ATZ
3	Ductal carcinoma, breast T1cN0M0, stage I	47	None	474	emtricitabine, tenofovir, raltegravir	RTV, ATZ
4	SCC, anus T2N0M0, stage II	47	None	NR	emtricitabine, tenofovir, raltegravir	RTV, DRV
5	Adenocarcinoma, prostate cT2bNXM0, GS 3+3, PSA 8.7, stage II	58	None	WNL	efavirenz, emtricitabine, tenofovir	RTV, LPV
6	Adenocarcinoma, prostate cT1cNXM0, GS 3+4, PSA 4.9, stage II	73	androgen deprivation	1105	raltegravir	RTV, DRV
7	Adenocarcinoma, prostate cT1cNXM0, GS 4+3, PSA 5.1stage II	69	androgen deprivation	536	abacavir, lamivudine	RTV, ATZ
8	Renal cell carcinoma, lateral chest wall, metastatic, stage IV	50	sutent	766	emtricitabine, tenofovir, efavirenz	RTV, ATZ
9	Arteriovenous fistula, dura mater	57	None	944	abacavir, lamivudine, raltegravir	RTV, LPV
10	SCC, tonsil T2N2bM0	53	cisplatin	956	emtricitabine, tenofovir	RTV, LPV
11	Primary CNS lymphoma, CNS	44	None	4	None	RTV, DRV
			doxil, cytoxan,			
12	NHL, neck and axilla, stage IV	53	vincristine, prednisone	57	abacavir, lamivudine	RTV
13a	NHL, pelvis, stage IV	53	None	120	abacavir, lamivudine	RTV, ATZ
13b	NHL, axilla, stage IV	55	None	39	abacavir, lamivudine	RTV, LPV
13c	NHL, temple, stage IV	56	None	87	abacavir, lamivudine	RTV, LPV
14	Primary CNS lymphoma, CNS	21	None	0	emtricitabine, tenofovir	RTV, DRV
15	Ductal carcinoma, breast T2N0M0, stage IIa	58	None	NR	emtricitabine, tenofovir	RTV, LPV
16	SCC, anus T1N0M0, stage I	43	5-FU, mitomycin C	547	abacavir, lamivudine	RTV, LPV
17	Primary CNS lymphoma, CNS	23	None	10	emtricitabine, tenofovir	RTV, DRV
18	Keloid scar, posterior scalp	47	None	WNL	zidovudine 300 mg, lamivudine 150 mg	NFV

Patients are uniquely identified by numbers, repeated treatments on a patient are distinguished by a letter after the number.

NHL = non-Hodgkin lymphoma

cT = clinical tumor, pT = pathological tumor, GS = Gleason score, PSA = prostate specific antigen

RTV = ritonavir, DRV = darunavir, ATZ = atazanavir, LPV = lopinavir, NFV = nelfinavir

WNL = Reported as within normal limits, NR = not reported

**Table 2:** Baseline data: patients not receiving concurrent protease inhibitor

#	Cancer diagnosis	Age	Concurrent systemic therapy	Baseline CD4	Non-PI HIV regimen	
1	SCC, cervix T4N1M0, stage IVa	29	cisplatin	189	None	
2	SCC, cervix, stage IIb	34	cisplatin	189	None	
3	Cholangiocarcinoma, abdomen pT3N1M0	53	xeloda	300	efavirenz, emtricitabine, tenofovir	
4	Adenocarcinoma, prostate TXNXM1, stage IV	48	None	399	None	
5	Meningioma, CNS	46	None	408	None	
6	Adenocarcinoma, prostate cT1cNXM0, GS	62	androgen	1047	None	
	3+4, PSA 20.6, stage II		deprivation			
7	NSCLC, brain met, stage IV	57	None	NR	None	
8a	DLBCL, brain met, stage IV	46	None	214	efavirenz, emtricitabine, tenofovir	
8b	DLBCL, brain met recurrence, stage IV	46	None	214	efavirenz, emtricitabine, tenofovir	
8c	DLBCL, testicular met, stage IV	46	None	214	efavirenz, emtricitabine, tenofovir	
9	Adenocarcinoma, prostate cT2aNXM0, GS	61	None	150	efavirenz, emtricitabine, tenofovir	
	3+4, PSA 1.1, stage II					
10	SCC, cervix, stage IIIb	57	cisplatin	116	None	
11	SCC, anal	49	mitomycin C and xeloda	450	efavirenz, emtricitabine, tenofovir	

Patients are uniquely identified by numbers, repeated treatments on a patient are distinguished by a letter after the number.

NHL = non-Hodgkin lymphoma, DLBCL = diffuse large B-cell lymphoma

cT = clinical tumor, pT = pathological tumor, GS = Gleason score, PSA = prostate specific antigen

NR = not reported

### Radiation treatment

For the 29 patients receiving radiation therapy, 15 patients were treated with definitive or adjuvant dose regimens (9 receiving PIs, 6 without PIs), while 14 patients received palliative radiation doses (9 receiving PIs, 5 without PIs). The exact definition of definitive/adjuvant versus palliative dose varied based on body site. Definitive/adjuvant dose was at least 5400 cGy for brain (conventional fractionation equivalent), 7000 cGy for head and neck, 5400 cGy for breast, 4500 cGy for pelvis, and 7800 cGy for prostate. Palliative doses also varied based on body site and disease histology, but were lower than definitive/adjuvant dose regimens.

### HIV medications and systemic chemotherapy

Systemic chemotherapy regimens for these two groups of patients are presented (Table 1 and 2). Of the 32 treatments for cancer (19 with PIs, 13 without PIs), 13 included systemic chemotherapy regimens (7 [37%] with PIs; 6 [46%] without PIs). 21 of the 29 patients were receiving HIV medications (17 [94%] with PIs; 4 [36%] without PIs; p = 0.001).

In the group receiving PIs, the most common PI was ritonavir (20 [95%]), followed by darunavir and lopinavir (7 [33%] each), atazanavir (5 [24%]), and only one [5%] patient received nelfinavir (Table 1 and 2).

### Toxicities

Follow-up and observed toxicities are presented in Table 3 and 4. The median follow-up of all patients was 18 weeks [IQR 8-30], but the follow-up for patients not taking PIs (median 13 weeks [IQR 5-18]) was much shorter than the follow-up for patients taking PIs (median 21 weeks [IQR 10-38]). The limited follow-up in the group not taking PIs prevented comparison of long-term toxicities.

**Table 3:** Radiation regimen, follow-up and toxicities in patients receiving concurrent protease inhibitor

#	F/U [weeks]	Region treated	Dose (fractionation) [cgy]	Complete RT regimen	Acute toxicity and CTC grade
1a	75	right breast	5800 (200)	yes	dermatitis 2, pruritis 1, hyperpigmentation 2, fatigue 1, pain 1
1b	0	pelvis	3600 (180)	no, prescribed 5400	fatigue 2, pain 2, nocturia 2, anorexia 1, proctitis 2
					fatigue 1, pain 1, nocturia and urinary
2	9	pelvis and left vulva	4500 (180)	yes	frequency 1, dysuria 2, proctitis 1, diarrhea
					1, mucosal drainage 1
3	36	right breast	5130 (270)	yes	fatigue 2, pain 3, dermatitis 1, hyperpigmentation 2
4	18	pelvis and anus	3000 (200)	yes	dysuria 1
5	82	prostate and SV	6720 (320)	yes	dysuria and nocturia and urinary frequency 2, anorexia 1, diarrhea 1, hematochezia 1
6	18	prostate and SV	8000 (200)	yes	nocturia and urinary frequency 2
7	7	prostate and SV	7800 (200)	yes	pain 1, dysuria and urinary frequency and incontinence 2, constipation 1, diarrhea 1
8	8	left lateral chest wall	3600 (300)	yes	dermatitis 1
9	38	brain	2000 (2000)	yes	none
10	25	head and neck	7000 (200)	yes	dermatitis 3, fatigue 3, dysphonia 1, xerostomia 2
11	21	brain	3000 (300)	yes	fatigue 2, pain 2, nausea 2, insomnia 2, anorexia 2, vomiting 2, ataxia 2
12	23	right neck and left axilla	3000 (200)	yes	fatigue 1, pain 1, dermatitis 1, dysgeusia 1, dysphonia 1, xerostomia 1
13a	147	right pelvis	3000 (250)	yes	fatigue 1
13b	13	right axilla and ulcerating skin lesion	3060 (180)	yes	dermatitis 2, drainage 3, pruritus 1
13c	7	right temple and subcutaneous skin lesion	3060 (180)	yes	dermatitis 2
14	19	brain	3000 (300)	yes	fatigue 1
15	86	right breast	6000 (200)	yes	dermatitis 1
					fatigue 1, pain 2, nausea 1, nocturia and
16	85	pelvis	5040 (180)	yes	urinary frequency 1, anorexia 2, proctitis 1,
					diarrhea 2, dermatitis 2
17	10	brain	3000 (300)	yes	altered mental status in intensive care throughout treatment
18	31	posterior scalp	1600 (400)	yes	none

Patients are uniquely identified by numbers, repeated treatments on a patient are distinguished by a letter after the number.

F/U = follow-up

**Table 4:** Radiation regimen, follow-up and toxicities in patients not receiving concurrent protease inhibitor

#	F/U [weeks]	Region treated	Dose (fractionation) [cgy]	Complete RT regimen	Acute toxicity and CTC grade
1	6	Pelvis	5400 (180)	yes	fatigue 1, nocturia 1, proctitis 2, gastrointestinal bleed 3, dermatitis 2
2	6	vaginal cuff brachytherapy	4500 and 2500 HDR (180 and 500 HDR)	yes	
3	18	Abdomen	5040 (180)	yes	fatigue 1, anorexia 1, nausea 1
4	13	thoracic spine	3000 (300)	yes	fatigue 1
5	29	Brain	5400 (180)	yes	pain 2, edema 2
6	44	prostate and SV	7800 (200)	yes	nocturia and urinary frequency and urgency 2, urinary retention 1
7	20	Brain	5300 (250 and 18 Gy gamma-knife boost)	yes	memory impairment 1, concentration impairment 1
8a	0	prostate and SV	7800 (200)	yes	dysuria and nocturia 2, urinary retention 1, constipation 1
8b	13	Pelvis	3780 and 1400 HDR (180 and 700 HDR)	no	fatigue 2, anorexia 1, dermatitis 2
8c	4	Pelvis	3000 and 1200 IORT (200 and 1200 HDR)	yes	fatigue 1, pain 1, nocturia 1, anorexia 1, proctitis 1, diarrhea 1
9	13	Brain	2400 (200)	yes	fatigue 2, pain 2,
10	5	brain (repeated)	2400 (200)	yes	fatigue 2, anorexia 1, constipation 1, dermatitis 1,
11	0	Testicles	2700 (180)	no, prescribed 4140	pain 1, constipation 1, dermatitis 1

Patients are uniquely identified by numbers, repeated treatments on a patient are distinguished by a letter after the number.

F/U = follow-up

HDR = high dose radiation

There were 64 acute toxicities in the group receiving PIs (30 grade 1, 30 grade 2, 4 grade 3). In the group not receiving PIs, there were 36 acute toxicities (23 grade 1, 13 grade 2). The median number of toxicities experienced per patient was not different between the groups (3 [IQR 1-7] with PIs; 3 [IQR 2-3] without PIs). Chi-square analysis of the distribution of severity did not find statistically significant difference in the severity of toxicities between the two groups (p = 0.38). One radiation treatment in each group was stopped early, but neither of these was secondary to toxicity (no grade 3 toxicities in either patient).

## Discussion

Our retrospective review of HIV-positive patients receiving radiation therapy found no increased toxicity in patients receiving concurrent PIs. The number and severity of toxicities experienced per patient were not found to be different in patients who were concurrently taking PIs compared to those who were not. There were differences in the baseline characteristics and medication regimens of the two groups. First, there were no cases of AIDS-defining malignancies in the group treated with PIs. This difference coincided with a difference in all HIV treatment and CD4 count. Significantly more patients in the non-PI group did not receive any medication to manage HIV, and significantly more patients in the non-PI group had CD4 counts below 500. This difference may reflect the efficacy of PIs and ART in controlling HIV, and a resulting decrease in opportunistic malignancies that has been observed with progressive generations of ART[9]. Although ART is typically initiated if the CD4 count is below 500, there are a number of other factors that contribute to the decision to initiate therapy, such as patient preference, adherence to prescriptions, and HIV strain. There was no association between CD4 count and adverse events.

There have been a number of case reports and small case series documenting seve re toxicities in HIV patients receiving radiation therapy. A meta-analysis of case reports and case series found severe toxicities in HIV patients receiving radiation therapy for Kaposi sarcoma and cervical carcinoma, but not in other malignancies[10]. Our results are in accordance with the only published study evaluating toxicities from interaction between PIs and radiation therapy [11]. Plastaras et al. reviewed 14 patients with concurrent PIs and 28 patients in the absence of PI, and found no difference in toxicity from radiation therapy. Although this group found no increase in toxicity from radiation therapy, the patient series was treated between 1993-2007 for the control group and 1997-2006 for the PI group. Inclusion of patients from this time period may have been reflected in the distribution of PIs and the distribution of malignancies treated. Nearly all patients in the Plastaras et al. study were treated with nelfinavir, three were treated with saquinavir (the oldest available PI), and one was treated with amprenavir (no longer available). 29 (69%) of 42 malignancies were AIDS-defining or strongly associated with HIV. These results may be limited by the baseline characteristics: AIDS-defining and HIV-associated malignancies are more heavily represented than in the current HIV+ population and PI regimens are evolving rapidly. Although not related to the years from which the patients were sampled, only 6 of the 14 patients from the PI group had documented CD4 count: one was <50, two were <200, and three <500. No association was observed between CD4 count and radiation toxicity, but the data is limited.

Our study characterizes the safety of radiation therapy combined with the newer generation of PIs in treatment of non-AIDS defining malignancies which are increasingly common in the era of improved ART. The series included only patients treated from January 1, 2009 onwards: of the 18 patients receiving PIs, 16 (89%) were receiving a dual-PI regimen; only two were taking a mono-PI regimen (one ritonavir and one nelfinavir). The case series included more malignancies not associated with HIV or AIDS (ductal carcinoma of the breast, renal cell carcinoma, cholangiocarcinoma, and meningioma), and two non-malignancies (dural AVM, and keloid scar) that were treated with radiation. Half of the patients in this case series received definitive or adjuvant radiation dose regimens (45-78 Gy). These patients were distributed equally in the group with PIs and in the group without PIs, and combination of definitive/adjuvant doses of radiation with PIs did not increase toxicities over definitive/adjuvant radiation doses alone. The present study more than doubles the reported number of patients treated with HIV PIs and radiation from 14 to 32.

The limitations of this study include the small size, short follow-up, heterogeneous nature of our cohort, and the differences between the control group and the PI treatment group. As discussed before, in addition to not taking PI, the control group also received less non-PI HIV medications and had a lower median CD4 count. The factors that underlie these two differences may confound the results. In addition, although we collected data on late toxicities, there was insufficient follow-up (21 weeks [IQR 10-38] with PIs, 13 [IQR 5-18] without PIs) to assess differences in late toxicities. Extended follow-up is necessary to determine the impact on long term toxicities. In addition, the majority of the cases received ritonavir combined with a second PI. Ritonavir does not inhibit Akt, which is a proposed mechanism of radiosensitization by PIs [27]. However, there are no published studies evaluating the radiosensitizing effect of darunavir, atazanavir, or lopinavir, which were used in combination with ritonavir by the majority of the patients. Prior studies on radiosensitization by PIs have not found a defining structural characteristic which would predict whether a PI will increase radiosensitivity. In spite of these limitations, this retrospective review provides valuable information about the acute toxicity of combining radiation with current PI therapies. Review of this contemporary series of patients did not find an increase in acute toxicity from the combination of the newest generation of HIV PIs and radiation therapy to treat diverse pathologies.

## Conclusions

Preclinical data has suggested that PIs used in the treatment of HIV may radiosensitize cancer cells, but case reports have suggested that PIs may exacerbate radiotoxicity in normal tissue. Review of a set of HIV-positive radiation therapy patients did not reveal increased toxicity in patients taking PIs during radiation therapy. Our cohort doubles the number of patients in the current literature on the acute safety profile of combining PIs and radiation therapy. These data suggest that clinical trials of PIs as radiosensitizers will not encounter increased acute toxicity.

## Competing interests

The authors declare that they have no competing interests.

## Authors' contributions

APS identified the HIV-positive patients receiving radiation treatment, performed the statistical analysis and helped draft the manuscript. JZ designed the protocol, collected clinical variables in review of the patient records and helped draft the manuscript. PTT and ML conceived of the study, designed the study and edited the manuscript. All authors read and approved the final manuscript.
